# Effect of physical activity interventions on quality of life in older adults: A protocol for systematic review and meta-analysis

**DOI:** 10.1097/MD.0000000000031801

**Published:** 2022-12-02

**Authors:** Nicola Lamberti, Fabio Manfredini, Jana Babjaková, Francesca Gallè, Kadri Medijainen, Christina Karatzaferi, Iuliia Pavlova, Yael Netz, Pablo Jesús López-Soto

**Affiliations:** a Department of Neuroscience and Rehabilitation, University of Ferrara, Ferrara, Italy; b Institute of Hygiene, Faculty of Medicine, Comenius University in Bratislava, Bratislava, Slovakia; c Department of Movement Sciences and Wellbeing, University of Naples Parthenope, Naples, Italy; d Lead of Physiotherapy curriculums, University of Tartu, Tartu, Estonia; e CREHP - Experimental Physiology Lab, DPESS, University of Thessaly, Trikala, Greece; f Department of Theory and Methods of Physical Culture, Lviv State University of Physical Culture named after Ivan Boberskyj, Lviv, Ukraine; g The academic College at Wingate, Israel; h Department of Nursing. Instituto Maimónides de Investigación Biomédica de Córdoba (IMIBIC), Córdoba, Spain; i Department of Nursing, Pharmacology and Physiotherapy. Universidad de Córdoba, Córdoba, Spain; j Department of Nursing, Hospital Universitario Reina Sofia de Córdoba, Córdoba, Spain

**Keywords:** COST, elderly, exercise, medicine, older adults, physical activity, quality of life, rehabilitation, wellbeing

## Abstract

Quality of life (QoL) has been acknowledged as a fundamental concept in the field of health and is favorably improved by physical activity (PA). This systematic review aims to assess the benefits and harms of different types of PA to improve health-related QoL in both community-dwelling and diseased older adults to provide a recommendation for the minimum amount of PA needed to obtain measurable QoL benefits.

**Methods and analysis:**

We will search MEDLINE, Cochrane Central Register of Controlled Trials, CINHAL, Epistemonikos, Web of Science and gray literature. Randomized controlled trials enrolling healthy or diseased older adults aged > 65 years, providing any kind of physical activity intervention and having quality of life as an outcome will be included. There will be no language restriction. Two independent reviewers will screen the papers, and a third reviewer will resolve the conflicts. The quality of the included studies will be assessed through the Risk of Bias 2.0 tool. Finally, data will be extracted to create proper meta-analyses of comparisons between the different kinds of physical activity interventions or to control groups.

**Ethics and dissemination:**

This review does not require approval from the Ethics Committee. The results will be disseminated in peer-reviewed journals and at international conferences; moreover, the findings will be shared on social media using an accessible language.

## 1. Introduction

There were 703 million persons aged 65 years or over in the world in 2019, with the number of older persons projected to double to 1.5 billion in 2050, and 1 out of 6 people in the world aged 65 years or over in that era.^[1]^ Particularly in Europe and Japan/Oceania, the proportion of people over 60 years old is expected to reach 50% in 2050.^[2]^ Population aging puts pressure on health systems, increasing the demand for care, services, and technologies to prevent and treat noncommunicable diseases and other conditions associated with old age.^[3]^ In this direction, globally active and successful aging has become a European Policy Perspective,^[4]^ and the 2030 Agenda for Sustainable Development released by the European Commission includes “Good Health and Wellbeing” within the main objectives.^[5]^ The translation of this approach into concrete social policies would mean the transformation of a wide range of policy arenas, such as the health dimension, toward more active interventions to prevent the causes of individual loss of function and loss of skills in old age.^[4]^ At the policy level, a redistribution of resources from acute to preventative health is essential by favoring strategies to promote health and wellbeing not only in older age but also before older age is reached.^[4]^

The first and furthest developed domain of successful aging is physical functioning.^[6,7]^ Maintaining physical function is an essential component of successful aging, and regular physical activity (PA) during the life span is a strong predictor of healthy aging.^[6–8]^ Decreases in muscle mass and muscle strength are related to aging processes as well as chronic diseases and unhealthy lifestyles (unbalanced nutrition, physical inactivity).^[9]^ The indicators of mobility performance and physical function are well known, and there is a consensus on measures and evaluation. For example, walking speed is an excellent marker of overall health and predicts the maintenance of physical function.^[6,9]^ In addition to these indicators of active aging, a deeper concept of successful aging derives from the widely used definition by the world health organization (WHO): as “the process of optimizing opportunities for health, participation and security in order to enhance the quality of life as people age,”^[10]^ considering that several cohort studies have reported that functional limitations are associated with lower quality of life in old age.^[6,11,12]^

Considering that the percentage of physically inactive older adults in Europe ranges from 5% to 29%,^[13]^ in North America is approximately 27,5%^[14]^ and bearing in mind that all adults aged ≥ 50 years, with or without chronic disease, gain health benefits by avoiding inactivity,^[15,16]^ several recommendations have been provided.^[17–19]^ The WHO published the new evidence-based guidelines for PA among older adults comprising at least 150 to 300 minute of moderate-intensity, or 75 to 150 minute of vigorous-intensity PA, per week, and 3 times a week multicomponent (e.g., balance, strength) PA.^[20,21]^ If on the 1 hand, this amount of PA seems to be easily attainable, on the other hand, only 23% of American healthy older adults reach the target,^[22]^ and this percentage decreases with female sex, older age, chronic diseases and the presence of lower limb symptoms.^[23]^ In addition, not all exercise regimens are universally effective, and interindividual differences in responses to PA exist.^[24]^

Quality of life (QoL) has been acknowledged as a fundamental concept in the fields of health and medicine.^[25]^ While there is no uniform definition of this concept, The WHO outlined 1 definition of QoL: “An individual’s perception of their position in the life in the context of the culture in which they live and in relation to their goals, expectations, standards and concerns.”^[26]^ The assumption is that irrespective of the objective level of the physical function or health condition of that individual, what counts is the personal perception of the individual.

In this direction, scientific literature encompasses more than 3000 reviews, systematic reviews and meta-analyses focusing on the effect of different modes of PA programs (e.g., aerobic exercise) on specific health outcomes (e.g., risk of falls) and quality of life in various groups of older adults (e.g., community-dwelling people), limiting the overall generalizability of the findings. For example, the benefits of resistance training in sarcopenia^[27]^ and the effects of aquatic physical exercise on neuropsychological factors in older people^[28]^ have been investigated. Quality of life is assessed in these reviews in addition to specific objective measures of sarcopenia or of neuropsychological factors, but rarely is considered the primary outcome.

In October 2021, the EU launched the COST Action CA20104 “Network on evidence-based physical activity in old age (PhysAgeNet)” to address the need to create “tailored” exercise programs that will fit the specific needs of the various and diverse aging populations.^[24]^ This action aims to embrace an evidence-based medicine (EBM) approach where conceptual challenges and pitfalls in basic research and clinical research on aging and physical activity can be identified and addressed. The principal unmet needs and gaps in research and practice for training older adults are the lack of research information needed for designing optimal, feasible and effective exercise programs for various target groups, including disabled, low-income and isolated older adults, and the lack of real-world condition studies over long periods.^[24]^

In this direction, this systematic review aims to collect evidence-based research and practice of physical activity in older adults from a wide-ranging perspective. The objective is to assess the benefits and harms of different types of PA when compared to a control group or another PA intervention to improve health-related

Health-related quality of life (HRQoL) in both community-dwelling and diseased older adults to provide a recommendation for the minimum amount of PA needed to obtain measurable HRQoL benefits.

## 2. Methods and analysis

This systematic review protocol was developed following the preferred reporting items for systematic review and meta-analysis protocols 2015 statement and checklist and Cochrane systematic review methodology.^[29,30]^ The protocol for the study was prospectively registered on the “International Prospective Register of Systematic Reviews” with the number CRD42022348068.

**Figure 1.**
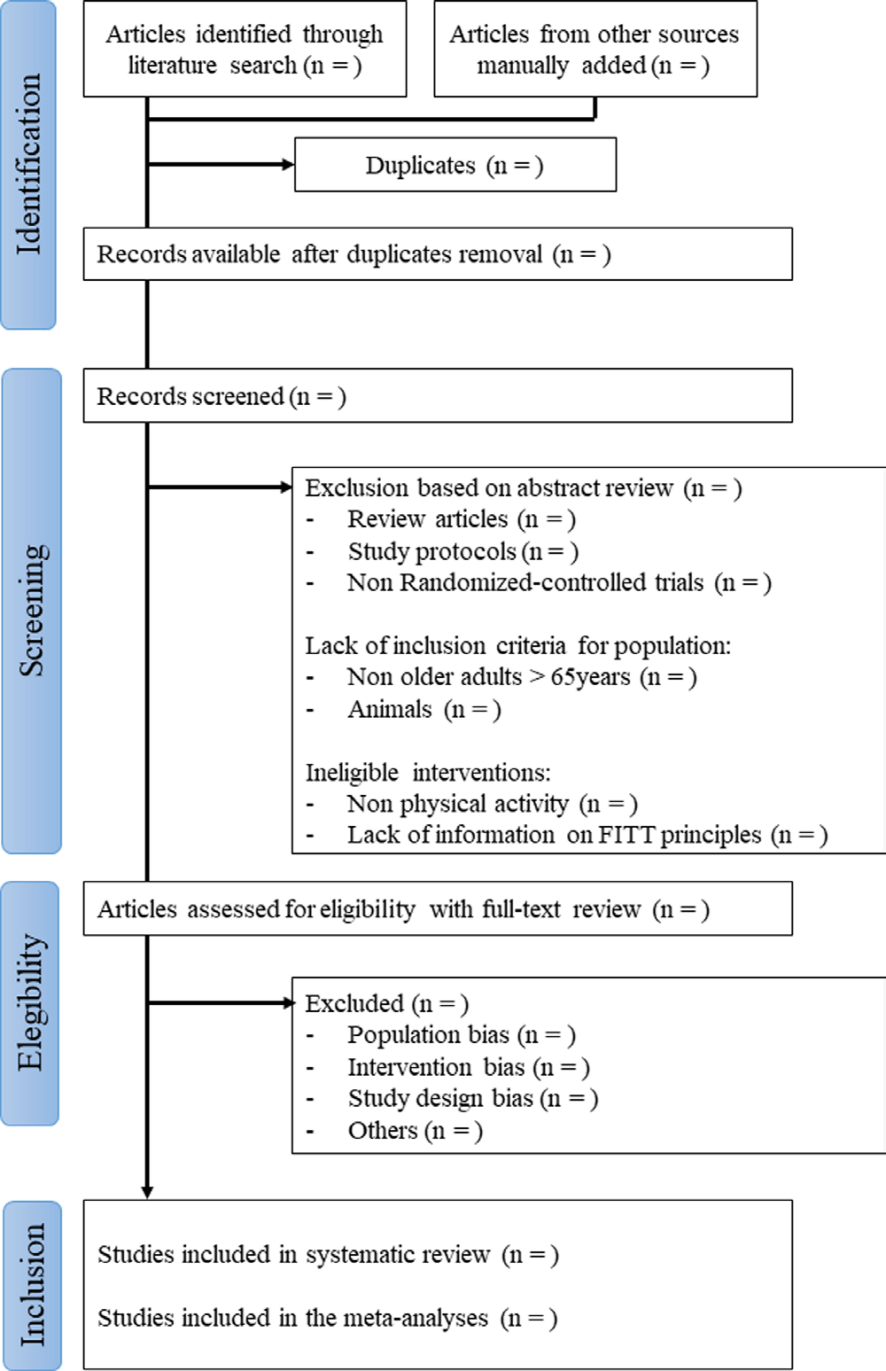
Flow chart of the systematic review.

The review design, including the selection process, is summarized in Figure 1.

### 2.1. Eligibility criteria

Eligibility criteria were determined through the PICOS framework as follows:

#### 2.1.1. Population.

Older adults aged > 65 years in a healthy condition or affected by chronic and non-communicable diseases. Studies including patients affected by life-threatening conditions or unable to walk or activities of daily living dependent will be excluded.

#### 2.1.2. Intervention.

We will screen any interventional study focused on the effect of any kind of PA. Interventions will include but will not be limited to physical activity interventions (defined as body movements that are produced by contraction of skeletal muscles and substantially increase energy expenditure), exercise training interventions, rehabilitation programs, tai-chi, etc. To draw a recommendation about the amount of physical activity needed, any exercise that cannot be resumed according to the frequency, intensity, time, and type principle will be finally excluded.

#### 2.1.3. Comparator.

All types of comparisons will be considered, including different types of physical activity interventions (e.g., aerobic vs resistance training), as well as against control groups at no interventions, acting as a control.

#### 2.1.4. Outcome.

HRQoL of the people, measured through validated questionnaires and scales, including but not limited to the Short Form-12, Short Form-36, EQ-5D, and WHO-QOL.

#### 2.1.5. Study.

To ensure good quality of the evidence, randomized controlled trials will only be included in the meta-analyses.

### 2.2. Inclusion and exclusion criteria

We will include studies of individuals aged 65 or older who are in either healthy conditions or affected by chronic diseases, and who were exposed to any kind of physical activity program (the intervention). The following Frequency, intensity, type, and time of exercise (FITT) (frequency, intensity, type, and time of exercise) principles will be applied for the program: frequency, at least 1 time per week; intensity, no limitations; type, no limitations; time, no limitations.

Studies involving other interventions associated with exercise but without a description of the FITT principles will be otherwise fully reviewed for possible inclusion.

Studies with participants in physical activity groups whose mean age is greater than 65 years will not be considered for inclusion, but only trials that encompass inclusion criteria for entry an age > 65 years.

### 2.3. Search strategy

A prior search was conducted in the MEDLINE database via PubMed to assess whether the research question of our review met the feasible, interesting, novel, ethical and relevant criteria. A preliminary search strategy for randomized controlled trials was then developed to be undertaken by 2 independent reviewers (NL and PJLS) in the following databases: MEDLINE, The Cochrane Library (Cochrane Database of Systematic Reviews, Cochrane Central Register of Controlled Trials (CENTRAL), Cochrane Methodology Register), CINHAL as the platform which collects nursing interventions, Epistemonikos, EMBASE and Web of Science (science and social science citation index). To minimize any publication bias, searches will also be undertaken on online gray literature (OpenGrey). We will also search clinical trial registries (Clinicaltrials.gov and WHO International Clinical Trials Registry Platform) for unpublished trials. Finally, we will take a look at EU-funded projects on the specific theme.

Systematic reviews already published on the specific theme will be screened to check if the articles included should also be considered for the purposes of this review.

There were no language restrictions for article inclusion. In the case of articles written in languages other than English, Spanish, Italian, Greek, Slovak, Polish, Ukrainian, Czech proper translation services will be employed.

Data for meta-analyses will be gathered through a careful review of the articles retrieved. Articles published from January 1^st^_,_ 1990 will be included. Upon completion of the review, we will undertake an additional search of all databases and registry platforms to ensure the inclusion of the most recent studies.

The main search terms will include “aged,” “older adults,” “physical activity,” “exercise” and “quality of life.” The search term related to the study design RCT will be entered into the databases that encompass its use (e.g., MEDLINE) to obtain more sensitive research. The full search strategy for MEDLINE is reported in Table 1.

**Table 1:** Search strategy for MEDLINE.

Population	(“Aged”[Mesh] OR “Aged, 80 and over”[Mesh] OR “elderly” [tiab] OR “old adults” [tiab] OR “older adults” [tiab] OR “senior*” OR “aging” OR “aging”)
*AND*	
Intervention	(“Exercise”[Mesh] OR “Exercise Therapy”[Mesh] OR “Physical Exertion”[Mesh] OR “Physical Fitness”[Mesh] OR “Sports” [Mesh] OR “Exercise Movement Techniques”[Mesh] OR “Exergaming”[Mesh] OR “Gymnastics”[Mesh] OR “Muscle Stretching Exercises”[Mesh] OR “Physical Conditioning, Human” [Mesh] OR “Preoperative Exercise”[Mesh] OR “Running”[Mesh] OR “Swimming”[Mesh] OR “Walking”[Mesh] OR “Warm-Up Exercise”[Mesh] OR “Circuit-Based Exercise”[Mesh] OR “Resistance Training”[Mesh] OR “High-Intensity Interval Training”[Mesh] OR “Blood Flow Restriction Therapy”[Mesh] OR “Rehabilitation”[Mesh] OR “physical activity” [tiab] OR “physical fitness” [tiab] OR “exercise” [tiab])
*AND*	
Outcome	(“Quality of Life”[Mesh] OR “QoL”[tiab] OR “HRQOL” [tiab] OR “quality of life” [tiab] OR “life quality” [tiab])
*AND*	
Study design	(“Randomized Controlled Trial” [Publication Type] OR “trial”[tiab] OR “randomized trial” [tiab] OR “randomized controlled trial” [tiab] OR “randomized study” [tiab] OR “randomized trial” [tiab] OR “randomized controlled trial” [tiab] OR “clinical trial”)

### 2.4. Data extraction and management

A study reviewer will be responsible for the extraction of data from all the intended databases (N.L.).

Upon the completion of the search in each database, all articles retrieved will be exported as “.ris” or “.nbib” files and imported into Rayyan, a web-based application for article screening.^[31]^ All duplicates will be removed by the same researcher using the proper Rayyan tool, considering that a duplicate is defined as an article that overlaps more than 90% with another manuscript.

At least 2 reviewers will work blinded and independently on study selection starting from an initial screening of title and abstract. When there was insufficient information in the abstract, the reviewers retrieved the full text of the article. Any conflict will be solved by another independent reviewer (P.J.L.S.).

In the following step, the full text of the selected articles will be retrieved, and the data of the studies will be extracted in a Microsoft Excel spreadsheet that was previously prepared. The database to be filled will contain: General characteristics (authors, year, journal); Population (number of patients of each group and characteristics including age, sex, medical conditions); Interventions (type of physical activity program and its characteristics according to the FITT principle, total duration, withdrawals, adherence); Outcomes (the instrument used to assess QoL, sample size, mean, standard deviations at baseline and all time points for all groups); and Notes (funding for the trial and notable declarations of interest of trial authors). When data are presented in graphs or are missing, we will contact the corresponding author to obtain them or use GetDate Graph Digitizer 2.26 to extract the data.

In the case of trials with multiple timepoints, the meta-analyses will be focused on the timepoint at the end of the physical activity intervention, as defined by the authors.

One review author (N.L.) will transfer data into Review Manager 5. To check for potential problems such as typographical errors in studies’ reports, the accuracy of data collection and manipulation, and data entry into Review Manager 5.4, 2 review authors will independently check if data are entered correctly. We will resolve disagreements by consensus. No study data will be extracted or analyzed by review members directly involved with the included studies.

We will extract the data by giving preference to; Change scores if both change and endpoint values are available and; Full intention‐to‐treat analysis; when this is not available, “as treated” or “per‐protocol” will also be considered.

All data extracted will then be formatted for the subsequent statistical analyses, which will be performed with MedCalc® Statistical Software version 20.110 and followings (MedCalc Software Ltd, Ostend, Belgium) and Review Manager (RevMan) Version 5.4.1 (by the Cochrane Collaboration, 2020).

### 2.5. Risk of bias

The risk of bias (RoB) in the included trials will be assessed using the Cochrane RoB 2 tool.^[29,32,33]^ The assessment is based on a set of 5 domains of possible biases. The 5 domains include randomization process, deviations from intended interventions, missing outcome data, measurement of the outcome and selection of the reported results. In each domain, several signaling questions are present for which we will provide any of the possible answers (“Yes,” “Probably yes,” “No,” “Probably no,” and “No information”). We will motivate the answers by making notes for each question. After this step, we will judge each domain according to the algorithm result as “Low risk of bias,” “Some concerns,” or “High risk of bias” for each outcome. The overall risk-of-bias judgement for each outcome will be the least favorable assessment across the domains. Considering that all the trials involve intervention based on physical activity, the blinding of study participants is not possible a priori, since interventions are described in the informative sheet necessary for ethical purposes. No trial will be excluded based on the risk of bias assessment.

### 2.6. Risk of overall bias in systematic reviews

The strength of the resulting evidence will be assessed through the Grading of Recommendations Assessment, Development and Evaluation tool.^[34]^ This tool resumes all the trials that are included in a specific meta-analysis, empowering the results obtained with an additional judgment on the level of evidence. This level can be “High” when the true effect lies close to that of the estimate of the effect, “Moderate,” when it is likely to be close, but there is still a possibility that it could be different, “Low” when it may be substantially different, or “Very low” when it is likely to be substantially different. These final evaluations came from the analysis of 7 domains, including RoB, imprecision, inconsistency, indirectness, publication bias, the magnitude of effect, dose–response gradient, and residual confounding.^[34]^ We will justify all decisions to downgrade the certainty of the evidence for each outcome using footnotes and make comments to aid the reader’s understanding of the review, if necessary. Two review authors will independently assess the certainty of the evidence.

### 2.7. Analysis strategy

Dichotomous data will be analyzed as risk ratios with 95% confidence intervals (CIs). Continuous data will be analyzed as the mean difference (MD) and 95% CIs when the studies use the same questionnaire or scale. When data are reported in different units (e.g., for different questionnaires), the standardized mean difference (SMD) will be used, together with their 95% CIs. Since the SMD implies a wider interpretation of the results (in absolute units), we may convert these measures into proportions (or percentages) so that the data are consistently presented for the outcome of interest.

Both MD and SMD will be pooled employing a fixed- or random-effects model according to the similarities or dissimilarities of the analyzed studies; for example, when data are reported in different units, a random-effect model will be used.

We will assess statistical heterogeneity by visual inspection of the forest plot to evaluate apparent differences in results between the included studies and using the I-squared (*I*^2^) and Chi-squared statistical tests.

The interpretation of the *I*^2^ statistic will be as recommended in Chapter 10 of the Cochrane Handbook for Systematic Reviews of Interventions^[35]^: “not be important” from 0% to 40%; “moderate” from 30% to 60%; “substantial” from 50% to 90% and “considerable” from 75% to 100%. We acknowledge that the importance of the *I*^2^ statistic depends on the magnitude and direction of effects and the strength of evidence for heterogeneity, which may be substantial when the number of studies is small. We will interpret the Chi^[2]^ test at a *P* value of 0.10 or less to indicate evidence of statistical heterogeneity. If we identify substantial heterogeneity, we will report it and investigate possible causes by following the Cochrane Handbook for Systematic Reviews of Interventions^[35]^ recommendations.

Publication bias, possibly derived from the selection or suppression of the results, will be assessed by examining the funnel plot and by conducting Egger’s test.^[36]^ First, a visual interpretation of the funnel plot for possible asymmetries will be performed. Then, if the result of Egger’s test (*P* < .100) suggests publication bias, we will apply the “trim-and-fill” method to identify and correct the funnel plot asymmetry.^[29,37]^

In trials with multiple arms, we will determine the intervention groups relevant for the present meta-analysis; if the characteristics of the intervention group will be similar according to the FITT principle, we will pool them to obtain a single pairwise comparison. For Randomized controlled trials with a crossover design, all measurements will be collected for both intervention and control groups (pre- and post-intervention/control), and data will be analyzed as parallel groups, with individual SDs for each intervention.

### 2.8. Missing data

In case of missing or unretrievable data (e.g., from a publicly available dataset), we will contact the corresponding author of the specific study up to 2 times at the provided addresses. If not received within 4 weeks, we will consider the data unobtainable. If this is not possible and the missing data are thought to introduce serious bias, we will explore the impact of including such studies in the overall assessment of results by a sensitivity analysis. Two imputation methods will be employed for all the outcomes: the “best worst case” scenario (assuming that all missing data in the intervention group are related to beneficial outcomes and all missing data in the control group are related to harmful outcomes) and the “worst best case” scenario, assuming that all missing data in the intervention group are related to harmful outcomes and all missing data in the control group are related to beneficial outcomes. For continuous outcomes, a “beneficial outcome” will be represented by the group mean plus 2 SDs, while the “harmful outcome” will be the group mean minus 2 SDs. We will consider for both cases all randomized participants in the denominator.^[38]^ For dichotomous outcomes (e.g., the number of withdrawals due to adverse events), we will calculate the withdrawal rate using the number of participants randomized in the group as the denominator. For continuous outcomes (e.g., mean change in questionnaires or scales), we will calculate the MD or SMD based on the number of participants analyzed at that time point. If the number of participants analyzed is not presented for each time point, we will use the number of randomized participants in each group at baseline. If possible, missing standard deviations will be computed from other statistics, such as standard errors, CIs, or *P* values. If standard deviations cannot be calculated, we will impute them (e.g., from other studies in the meta-analysis).

### 2.9. Analysis plan

Considering the impossibility of a priori defining the types of interventions, comparators, outcomes and clinical conditions of the subjects of the included study, we plan to perform more subanalyses.

The principal analysis will be carried out in the global population to define whether any physical activity intervention will be superior to inactivity in the entire population of older adults, despite any clinical condition they may have.

Second, a pool of secondary analyses will be completed by classifying the population according to the healthy condition or disease category they are affected by, comparing the different types of physical activity (e.g., aerobic vs resistance training) and so on, investigating all the possible combinations of these factors to make the review as complete as possible.

### 2.10. Data synthesis

We will undertake meta-analyses only if this is meaningful (e.g., if participants, interventions, comparisons, and outcomes are judged to be sufficiently similar to ensure a clinically meaningful answer). If the implementation of a meta-analysis is not possible, we will use alternative synthesis methods, such as the summary of effect estimates (e.g., median, interquartile range with box-and-whisker plots).

We will follow the guidelines of the Cochrane Handbook for Systematic Reviews of Interventions to interpret the synthesis results and communicate the conclusions of the review effectively.^[29]^ We will focus on distinguishing a lack of evidence of an effect from a lack of an effect. We will base our conclusions on findings from the quantitative or narrative synthesis of included studies for this review. We will consider any statistical heterogeneity when interpreting results, particularly when a variation in the direction of effect is present.

Finally, we will provide an overall conclusion determining the amount of physical activity needed (in relation to the FITT principle) to obtain significant improvements in QoL. In addition, we will suggest priorities for future research and outline the remaining uncertainties in the area.

## 3. Conclusion

In conclusion, understanding the amount of physical activity necessary to obtain significant improvement in HRQoL is important in the elderly, as it may provide exercise options for a better management of the older adult population with and without chronic diseases. The meta-analyses conducted in the entire population and in the different subsamples can further support previous findings and improve our understanding and recommendations for the management of PA in this population.

## 4. Ethics and dissemination

This review does not require approval from an Ethics Committee. The results will be disseminated in peer-reviewed journals and at international conferences; moreover, the findings will be shared on social media using an accessible language.

## Acknowledgments

This publication is based upon work from COST Action CA20104 - Network on evidence-based physical activity in old age (PhysAgeNet), supported by COST (European Cooperation in Science and Technology). https://www.cost.eu/, https://physagenet.eu/

## Author contributions

**Conceptualization:** Nicola Lamberti, Fabio Manfredini, Jana Babjaková, Christina Karatzaferi, Pablo Jesús López-Soto.

**Data curation:** Nicola Lamberti, Fabio Manfredini, Jana Babjaková, Francesca Gallè, Kadri Medijainen, Iuliia Pavlova, Yael Netz.

**Formal analysis:** Nicola Lamberti, Fabio Manfredini, Jana Babjaková, Francesca Gallè, Kadri Medijainen, Iuliia Pavlova.

**Funding acquisition:** Fabio Manfredini, Yael Netz.

**Investigation:** Fabio Manfredini, Pablo Jesús López-Soto.

**Methodology:** Nicola Lamberti, Fabio Manfredini, Jana Babjaková, Francesca Gallè, Kadri Medijainen, Christina Karatzaferi, Iuliia Pavlova, Yael Netz, Pablo Jesús López-Soto.

**Project administration:** Nicola Lamberti, Fabio Manfredini, Christina Karatzaferi, Pablo Jesús López-Soto.

**Resources:** Iuliia Pavlova, Yael Netz.

**Software:** Kadri Medijainen.

**Supervision:** Nicola Lamberti, Christina Karatzaferi, Yael Netz, Pablo Jesús López-Soto.

**Visualization:** Iuliia Pavlova.

**Writing – original draft:** Nicola Lamberti, Fabio Manfredini, Jana Babjaková, Francesca Gallè, Kadri Medijainen, Christina Karatzaferi, Iuliia Pavlova, Yael Netz, Pablo Jesús López-Soto.

**Writing – review & editing:** Nicola Lamberti, Fabio Manfredini, Jana Babjaková, Francesca Gallè, Kadri Medijainen, Christina Karatzaferi, Iuliia Pavlova, Yael Netz, Pablo Jesús López-Soto.
